# Effectiveness of Attentional Bias Modification Combined With Cognitive Behavioral Therapy in Reducing Relapse Risk and Cravings in Male Patients With Alcohol Use Disorder: A Quasi‐Randomized Controlled Trial

**DOI:** 10.1002/npr2.70002

**Published:** 2025-02-05

**Authors:** Yoshifumi Amano, Kohei Koizumi, Hirokazu Takizawa, Shota Tasaka, Toyohiro Hamaguchi

**Affiliations:** ^1^ Narimasu Kousei Hospital Itabashi Ward Tokyo Japan; ^2^ Department of Rehabilitation, Graduate School of Health Science Saitama Prefectural University Koshigaya City Saitama Prefecture Japan

**Keywords:** alcohol relapse risk scale, alcohol use disorder, attention bias, cognitive behavior therapy, cravings

## Abstract

**Background:**

Alcohol use disorder (AUD) is characterized by severe dependence on alcohol, poor impulse control, and heightened attention to alcohol‐related cues. Attention bias modification (ABM) retrains individuals to distract attention from alcohol‐related cues. This study investigates the effect of combining ABM with cognitive behavioral therapy (CBT) to reduce relapse risk and cravings in male patients with AUD.

**Methods:**

A quasi‐randomized controlled trial was conducted among male inpatients diagnosed with AUD. Participants were divided into an intervention group receiving ABM in addition to CBT and a control group receiving CBT with a placebo intervention. The primary outcomes—risk of relapse and craving levels—were measured using the Alcohol Relapse Risk Scale (ARRS) and a visual analog scale (VAS), respectively. Participants underwent weekly sessions over 6 weeks, and outcomes were analyzed using generalized linear models (GLMs).

**Results:**

The analysis did not reveal significant interactions between the intervention group and time for ARRS scores and craving levels. Both groups experienced a reduction in relapse risk and cravings. However, there was no significant difference between the ABM + CBT and CBT‐only groups.

**Conclusions:**

Although the combined ABM and CBT intervention did not result in statistically significant reductions in relapse risk and cravings compared to CBT alone, the overall reduction in these outcomes in both groups highlights the effectiveness of CBT in treating AUD. Future studies should use naturalistic settings and tailor the intervention to individual cognitive profiles.

## Abstract 2

This quasi‐randomized controlled trial investigated a novel combined approach of attention bias modification (ABM) with cognitive behavioral therapy (CBT) for male inpatients with alcohol use disorder (AUD). Both the CBT + ABM intervention and CBT‐alone groups showed substantial improvements in relapse risk and craving levels over the 6‐week treatment period, demonstrating the therapeutic potential of integrating attention retraining with conventional treatment. These findings support the viability of both treatment approaches while suggesting opportunities for future research to optimize combined interventions in naturalistic settings.

## Graphical abstract

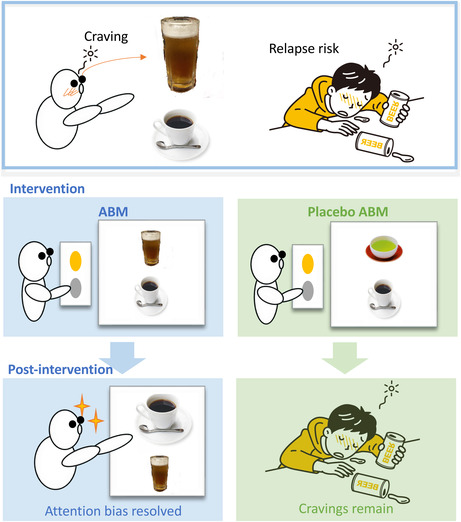

## 1 Introduction

Alcohol has a pharmacological effect on central nervous system (CNS) depression. Repeated drinking increases alcohol consumption, leading to physical and psychological dependence on alcohol. Neuropsychological evidence suggests that patients with alcohol use disorder (AUD) have specific impairments indicative of frontal lobe dysfunction [1]. The prefrontal cortex inhibits thoughts and behaviors when performing complex tasks [2]. When this region is impaired or dysfunctional, it becomes difficult to control alcohol intake. Additionally, binge drinkers (defined as individuals consuming five or more drinks within a 2‐h period at least once in the past 2 weeks) show increased brain wave amplitude when attention is directed toward alcohol compared to healthy individuals [3]. These findings suggest that heavy drinkers have impairments or dysfunctions in the frontal lobe functions that inhibit alcohol consumption and neural activities related to paying attention to alcohol [4].

This study excluded women from participation owing to sex differences in alcohol metabolism. For example, Colzato [5] showed that women with higher circulating levels of sex hormones, including Estradiol (E2), have weaker psychological impulse inhibition than men. In Japan's Health 21 initiative, the “amount of drinking that increases the risk of lifestyle‐related diseases” is defined as 40 g or more for men and 20 g or more for women [6]. Women have a lower body water content than men owing to physical differences, making it easier for blood alcohol concentrations to rise [7]. In addition, there are sex differences in the ability to metabolize acetaldehyde due to differences in liver size (1.5 kg in men, 1.3 kg in women) [8]. There are also sex differences in dopamine D1 receptors, with men showing higher receptor concentrations in the nucleus accumbens than women [9]. Excitatory D1 receptors in the nucleus accumbens are involved in reinforcing the properties of addictive substances [10]. In studies on impulsive behavior, men showed localized brain activation during successful inhibition, while women showed greater brain activation during inhibition failure compared to success [11]. Based on these findings, it is expected that choosing intervention methods tailored to the characteristics of men and women to attenuate responses to alcoholic stimuli will lead to more efficient treatments.

Cravings are a characteristic symptom of AUD. A craving for alcohol is defined as an uncontrollable desire to consume a substance and has been studied in relation to alcohol use disorders and relapse [12]. Previous studies have reported that among patients with AUD with high and low cravings, those with high cravings find it difficult to resist the urge to drink after being presented with alcohol images [13]. Cravings in patients with AUD are associated with the risk of relapse [14]. Specifically, in patients with AUD who have just completed detoxification, the relationship between cravings and attention is prominent and has been reported to correlate with a decrease in dopamine D2 receptor availability and dopamine in the ventral striatum [15]. The decreased availability of dopamine D2 receptors in the ventral striatum of recently detoxified patients with AUD is associated with the increased functional activation of the anterior cingulate cortex and adjacent medial prefrontal cortex induced by alcohol‐related and neutral images. These brain regions are associated with attention to salient stimuli, and the stimulus‐induced activation of these regions is considered a predictive factor for relapse risk in detoxified patients with AUD [16]. It can take time for patients with AUD to recover from alcohol dependence [17] and relapse is considered part of the recovery process from alcohol and other drug dependencies. However, there are risks associated with relapse; for example, morbidity and mortality from alcoholic liver disease are much higher in people who relapse compared to abstainers [18]. Therefore, relapse prevention is important.

Biased cognitive processing of alcohol‐related stimuli is a confirmed trigger for alcohol‐seeking behavior and relapse in patients with AUD [19]. Patients with AUD are said to have an attentional bias (AB) toward alcohol. AB is selective attention to the target substance that is hypothesized to have a causal relationship with the development and maintenance of drug abuse and addiction [20]. High‐frequency drinkers have an AB that directs their attention to words associated with drinking for longer periods than low‐frequency drinkers [21]. Thus, AB is likely to trigger pathological drinking in patients with AUD.

Attentional bias modification (ABM) has been used to control drinking behavior in patients with AUD. The dot‐probe task is a frequently used intervention method [22]. In AB training, two stimulus images (one substance‐related and one substance‐unrelated) are displayed simultaneously on the screen. Next, both stimulus images disappear, and a probe appears behind the substance‐unrelated stimulus. The patients are instructed to quickly identify the probe's position using a keyboard or other input device. Consequently, patients learn to direct their attention toward substance‐unrelated and away from substance‐related stimuli. Thus, AB toward substance‐related cues is retrained. When patients with AUD undergo a visual probe task, their response to avoiding alcohol‐related stimuli becomes faster, and relapse occurs more than 1 month later than for the placebo group [23]. Conversely, Field et al. [24] found that AB training alone does not result in a long‐term reduction in drinking behavior. This study conducted an AB training set to avoid alcohol stimuli and investigated participants' alcohol consumption after training but found no difference in consumption compared to the control group. This suggests an insufficient acquisition of alternative behaviors. The results also showed that the effect of reducing drinking behavior was not maintained over the long term, suggesting that AB training alone may not lead to a long‐term reduction in drinking behavior. Therefore, it may be useful to combine it with other techniques.

Cognitive behavior therapy (CBT) is used to treat alcohol and drug dependence [25]. While CBT is commonly used to treat depression, anxiety disorders, and other mental disorders, it is based on the idea that emotions and behaviors are caused by a person's thoughts rather than external stimuli—people, situations, or events. Patients with AUD often use alcohol to cope with cognitive distortions, problematic events, or emotions related to other mental health disorders [26]. CBT for use disorders has been reported to be highly effective in supporting the acquisition and modification of coping skills [27].

A study on anxiety disorders demonstrated that CBT and ABM complement each other by targeting different cognitive aspects of anxiety [28]. ABM uses a bottom‐up approach to control conscious behavior and address unconscious aspects, whereas CBT uses a top‐down approach. Amano et al. [29] reported that cravings in patients with AUD are related to AB reaction time (AB‐RT) and derived the regression equation: Craving = (3.1 × AB‐RT) + (−32.2 × gender) + (−0.1 × γ − GTP) + 36.4. They further reported that Craving is also related to relapse risk (Alcohol Relapse Risk Scale: ARRS) and derived the regression equation: Craving = (1.9 × ARRS total score) + (−38.0 × gender) + (−0.1 × γ‐GTP) + 26.7. Based on this report, this study predicts that reducing AB‐RT and relapse risk will also decrease cravings. In other words, this study tests the idea that if biased AB toward alcohol could be modified, in addition to CBT—traditionally used to modify cognition toward drinking in patients with AUD—it could further reduce cravings and relapse risk.

**FIGURE 1:**
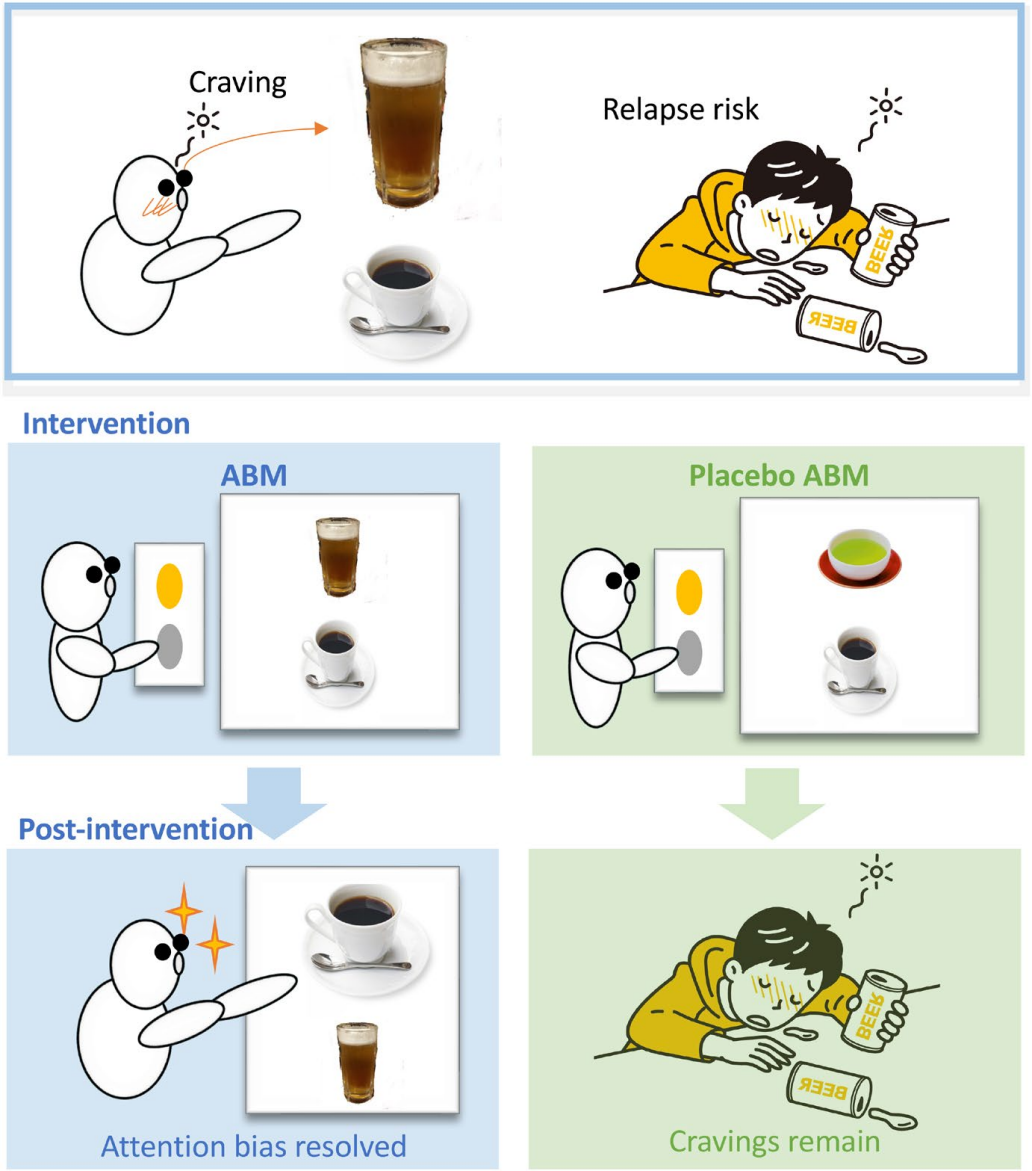
Hypothesis and intervention framework of the study. The study hypothesizes that patients with AUD have an attentional bias (AB) toward alcohol, which influences cravings and relapse risk. Patients with AUD undergoing a CBT program were divided into an intervention group receiving ABM and a control group receiving placebo ABM. It was hypothesized that the intervention group would experience a greater reduction in craving and relapse risk compared to the control group.

This study targeted male patients with AUD who had undergone a CBT program and aimed to verify the following hypotheses: (1) Adding ABM intervention reduces the risk of relapse, and (2) ABM intervention is more effective in lowering cravings for alcohol compared to the ABM placebo. The results of this study were intended to develop a new rehabilitation program aimed at lowering cravings and relapse risk in patients with AUD when ABM was conducted in addition to CBT (Figure 1).

## 2 Methods

### 2.1 Research Design

This interventional study was designed as a quasi‐randomized controlled trial in which participants were alternately assigned to either the intervention or control group.

### 2.2 Participants

All participants were previously diagnosed with alcohol dependence by physicians using ICD‐10 diagnostic criteria. After completing the initial 2‐week hospitalization period and withdrawal management, potential participants were provided with study information. Following informed consent, AUDIT was administered to obtain baseline severity scores. This systematic approach ensured that the participants were medically stable and had completed acute withdrawal before study enrollment.

**FIGURE 2:**
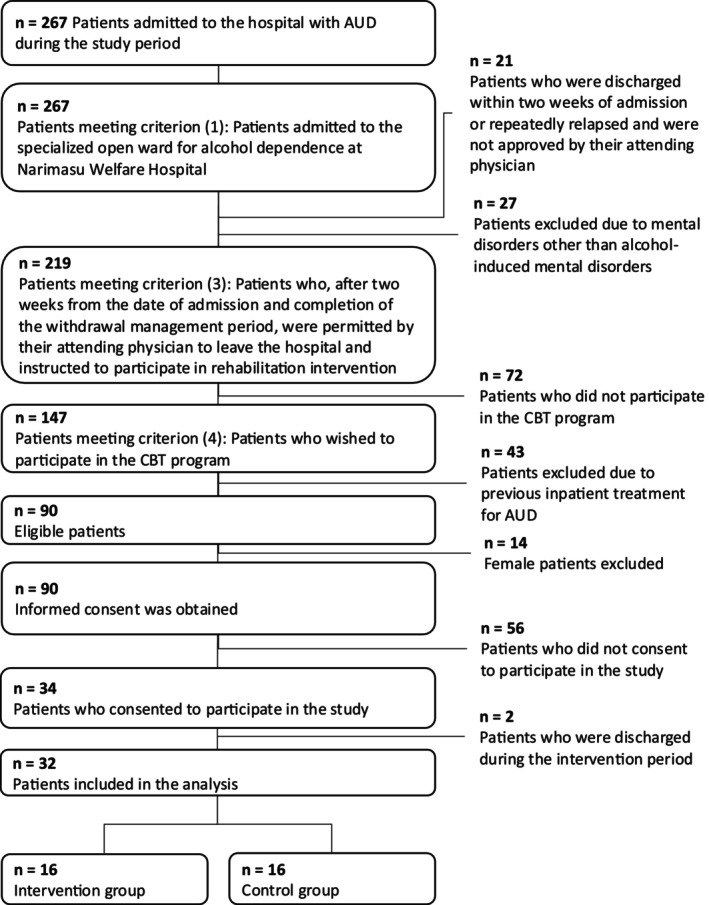
Flow diagram of patient progression through phases of the trial. Out of 267 patients admitted during the study period, 90 met the inclusion and exclusion criteria and were informed about the study. Thirty‐four patients consented to participate, with 32 completing the study after two withdrew. Sixteen participants were assigned to each group (intervention and control).

The eligibility criteria for participants were as follows: (1) patients with AUD hospitalized in the specialized open ward for alcohol dependence at Narimasu Kousei Hospital; (2) those who had completed 2 weeks since admission, concluded withdrawal management, and received permission from their attending physician for outside visits and rehabilitation intervention; and (3) those who wished to participate in the CBT program. The exclusion criteria were as follows: (1) those with mental disorders other than alcohol‐induced mental disorders, (2) those who had previously received inpatient treatment for AUD, (3) female patients, and (4) those who were discharged for any reason during the intervention (Figure 2).

Inpatient treatment included medication, occupational therapy, psychotherapy, and participation in self‐help groups. The patients provided informed consent to receive inpatient care. All the participants received inpatient treatment.

### 2.3 Protocol

**FIGURE 3:**
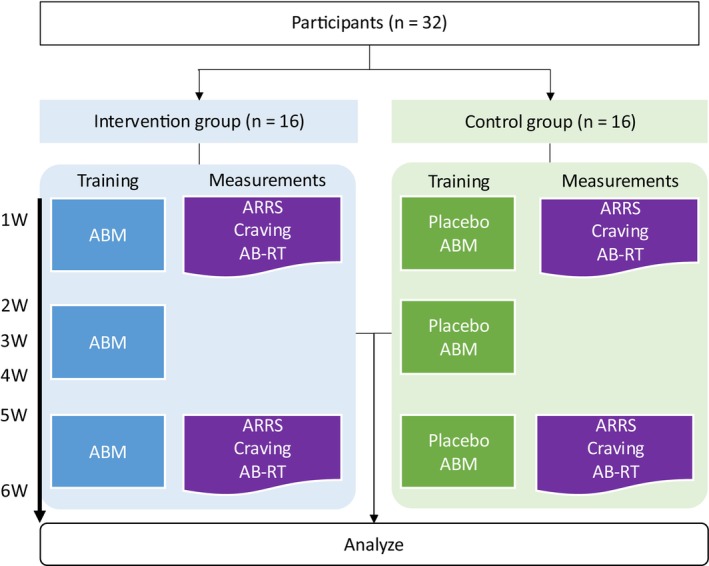
Intervention study procedures. The intervention group performed ABM weekly, while the control group received placebo ABM weekly. Both groups were assessed for ARRS, Craving, and AB‐RT in the first and sixth weeks.

This study was conducted in the ward of the Tokyo Alcohol Medical Center at Narimasu Kousei Hospital. Participants who met the eligibility criteria were given a written explanation of the study content, and their consent for participation was obtained. The participants were subsequently assigned to either the intervention or control group (without knowing which group they were in), and attentional bias measurements and psychological evaluations were conducted for approximately 30 min. Psychological tests were conducted during the first and sixth weeks, and AB measurements were obtained after the psychological tests (Figure 3). AB training was conducted weekly. Participants also received 80‐min CBT sessions once a week for 6 weeks starting the first week.

### 2.4 Intervention

#### 2.4.1 Attentional Bias Measurement

The ABM trainer application software (Ideoquest Co. Ltd., 2016, Tokyo, Japan) was installed on a personal computer (Dell Inspiron 14 5490) and used for AB measurement. A 14‐in. LCD color display was used for stimulus presentation. The participants were asked to select two images displayed on the screen using the ABM trainer's button‐type input device. The distance between the participant's face and the display was approximately 45 cm.

After the two images were presented by the ABM trainer, a probe stimulus was displayed, and the computer measured the time it took for the participant to press the input button. The reaction time was used as an indicator of AB. There were 128 trials for evaluating AB, and data with reaction times below 200 ms or above 2000 msec were excluded as erroneous responses [30]. The average reaction time (average correct response time) when selecting images of non‐alcoholic beverages was measured for each participant.

AB intervention studies have reported that effects are obtained when the number of trials exceeds 800 [31, 32]. For AB training, the number of trials in one session was set to 128, to exceed 800 trials over six sessions [33].

#### 2.4.2 Stimuli and Tasks

**FIGURE 4:**
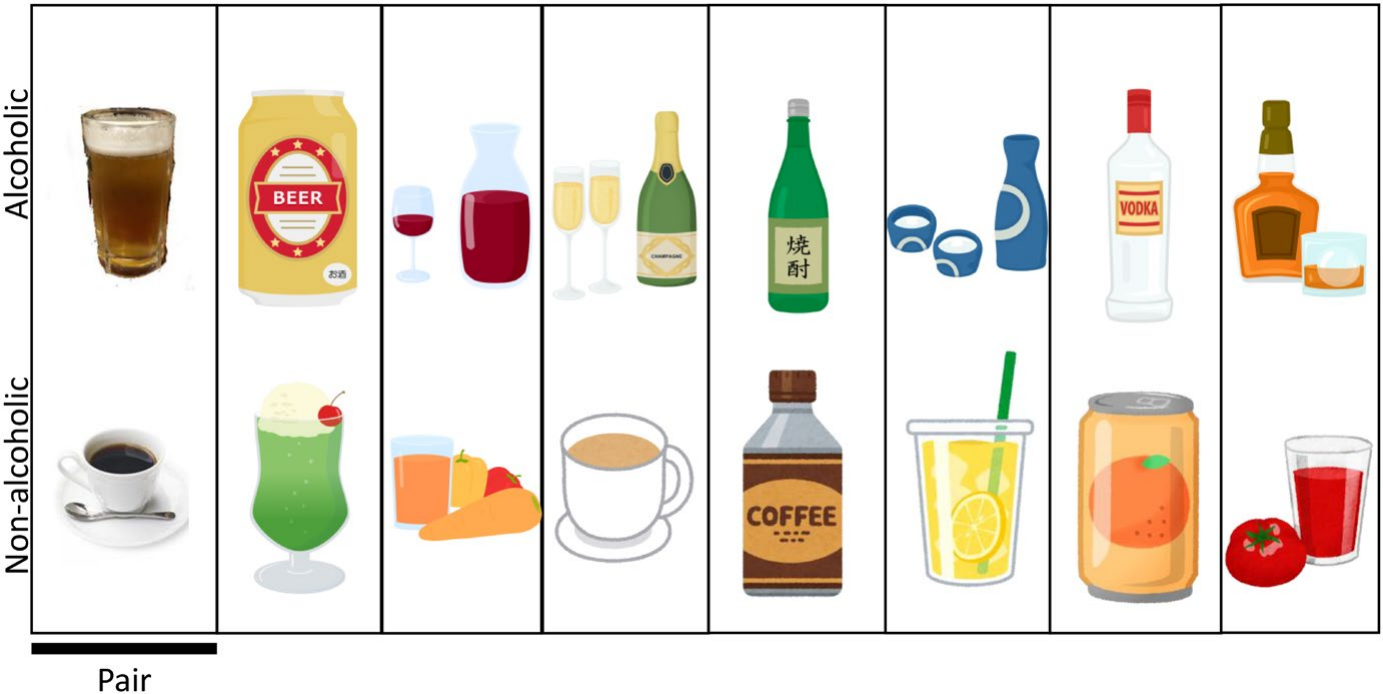
Paired images of alcoholic and non‐alcoholic beverages used in ABM. This figure displays the images used in ABM. In 128 trials, alcoholic and non‐alcoholic images were paired randomly either at the top or bottom. The images are blurred here, but unaltered images were used in the trials.

Images of commercially available alcoholic and nonalcoholic beverages were used to measure AB. Images of alcoholic beverages included beer poured into glasses and canned chu‐hai packages, whereas nonalcoholic beverages included images of bottled tea and coffee in cups. Eight pairs of images from the alcoholic and non‐alcoholic groups were prepared. Images were presented simultaneously in two vertically arranged 256 × 256 pixel images (Figure 4).

The training and placebo training for the training block were created based on a previous study [32]. The task involved selecting non‐alcoholic images from two vertically arranged images (including alcoholic and non‐alcoholic images) and pressing two input buttons. The eight types of stimulus images were randomly presented 128 times. In the intervention group's training, 90% of the trials presented the probe behind non‐alcoholic images (training attention away from alcohol), while 10% presented the probe behind alcoholic images. For the control group's placebo training, probe placement was more balanced: 60% behind non‐alcoholic images and 40% behind alcoholic images. This differential ratio was designed to create stronger attentional bias modification in the intervention group compared to the control group.

**FIGURE 5:**
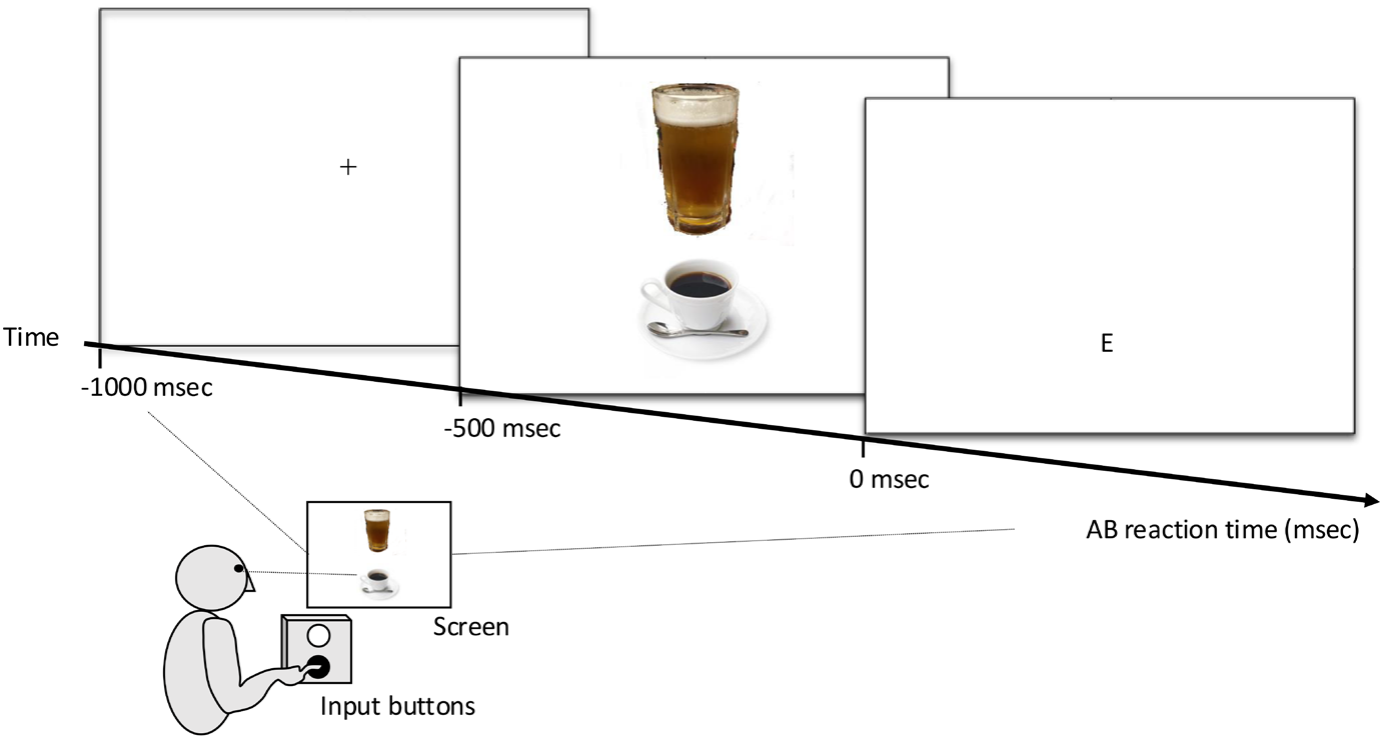
Measurement of attentional bias. In 128 trials, images of alcoholic and non‐alcoholic beverages were presented randomly either at the top or bottom (8 pairs of images). Images were displayed in a size of 3.75 cm by 5 cm. Participants were instructed to select the probe stimulus using a button. The “E” represents “Enter”. The response time (AB‐RT) to identify the images and select the probe stimulus was calculated. Trials with AB‐RT < 200 msec or > 2000 msec were excluded as erroneous responses.

The participants were tasked with select images from combinations of eight types of images. A focal point was presented in the center of the LCD display for 500 ms, and two target images for the participants to select from were subsequently presented for 500 ms. Participants were asked to select the non‐alcoholic image after the target images were presented and to press the corresponding button (Figure 5).

#### 2.4.3 Cognitive Behavioral Therapy

A CBT workbook was created by translating the Coping Skills Training created by Monti et al. [34] into Japanese and partially modifying the notation to suit Japanese people (Appendix S1). An 80‐min program using this workbook was implemented for the participants. Each program cycle had approximately 10 participants [35] (Appendix S2).

The CBT program consisted of six structured sessions. Session 1 discussed the advantages and disadvantages of both drinking and abstinence. Session 2 addressed identifying and understanding personal drinking triggers. Session 3 explored high‐risk situations that provoke alcohol cravings and developed specific coping strategies. Session 4 utilized cognitive restructuring through thought records to modify drinking‐related cognitions. Session 5 implemented role‐playing exercises to practice refusal skills when offered alcohol. Session 6 focused on developing concrete action plans and identifying specific support resources for managing imminent drinking risks.

The program was conducted according to the Coping Skills Training Manual [34], and the implementation method was described in the text to minimize differences between implementers. Occupational therapists, certified psychologists, and nurses conducted the program.

### 2.5 Outcomes

The primary outcomes of this study—relapse risk and craving—were measured using the Japanese version of the Alcohol Relapse Risk Scale [36] and VAS [37], respectively. The secondary outcome was the attention bias reaction time (AB‐RT). Basic information about participants, including age, length of hospital stays, and γ‐GTP value as an indicator of liver function, was collected from medical records. The number of relapses that occurred from the start of the intervention until discharge was recorded. The Alcohol Use Disorder Identification Test (AUDIT, WHO) [38] was used to evaluate AUD.

As this study investigated whether the combination of AB modification training and CBT reduces the risk of relapse, the total ARRS score was adopted as the measure of the primary outcome. The ARRS is a self‐administered, 32‐item questionnaire that quantitatively evaluates the risk of relapse in patients with AUD. The questions were classified into six subscales (factors): *Vulnerability to Stimuli*, *Emotional Problems*, *Impulsivity to Drink*, *Lack of Alcohol Harm Recognition*, *Positive Expectations for Drinking*, and *Strength of Disease Awareness*. The risk of relapse was evaluated by the total score of five items (ARRS Total) excluding *Strength of Disease Awareness*, with higher scores indicating a higher risk of relapse.

The strength of alcohol craving (*Craving*) was evaluated using the VAS. The VAS used a 100 mm line with extreme values of 0 and 100, and the position of the marked line was scored. The scores ranged from 0 to 100. The VAS was set with 0 = no craving at all and 10 = strongest imaginable craving, and the subjective strength of the average craving over a week was evaluated [37]. The VAS is commonly used because of its ease of use and simple numerical scoring, and it can be treated as a continuous variable in statistical analysis [39].

The AUDIT is a screening test for problem drinkers developed by the WHO and is used as a tool for early detection of and intervention in drinking problems in many countries. The AUDIT consists of 10 questions, and the degree of drinking problems is evaluated by the total score of each item (maximum 40 points). The cut‐off point of AUDIT was based on the Standard Health Checkup/Health Guidance Program (Revised Version) used in specific health guidance, with increasing risk scoring 8–14 points, and those with higher scores considered at risk of dependence.

For psychological indicators, the Japanese version of the Profile of Mood States 2 (POMS2) Short Form [40, 41], was used. POMS2 is a psychological test that can simultaneously measure seven factors: anger‐hostility, fusion‐bewilderment, depression‐dejection, fatigue‐inertia, tension‐anxiety, vigor‐activity, and friendliness. In the POMS2 Short Form, the number of question items was reduced from 65 to 30, thereby reducing the burden. The questionnaire expresses moods classified into seven scales with 30 words, and participants were asked to choose one of five levels (0 to 4 points) from “Not at all” (0 points) to “Extremely” (4 points).

### 2.6 Estimation of Required Number of Participants for Analysis

The sample size for this study was determined using Gpower. Based on Jones and Sharpe's [42] study, the effect size was set at 0.8, with an α error of 0.05 and a power of 0.8. The total sample size was calculated to be 26. The overall target number of participants was set at 30 because we anticipated a 10% dropout.

### 2.7 Quasi‐Randomization Procedure

In this study, the participants were alternately assigned to the intervention or control group in the order of their participation. Participants were not informed of the group to which they were assigned. The assignment was conducted by the study implementer.

### 2.8 Statistical Analysis

This study compared the intervention and control groups using the ARRS Total and Craving (VAS) as outcomes for the combined intervention of ABM and CBT. To test Hypothesis (1) that the combined intervention of ABM and CBT reduces the risk of alcohol relapse, a Generalized Linear Model (GLM) was used. The response variable was ARRS Total, with group (indicating intervention or control group) and time (indicating intervention period) as explanatory variables, and age and initial ARRS Total value as covariates. Akaike's Information Criterion (AIC) was used for model fit.

For the second analysis, a GLM was used to test hypothesis (2) that the combined intervention of ABM and CBT reduces alcohol cravings. The response variable was craving (VAS), with group and time as explanatory variables and age and initial craving value as covariates. For the first supplementary analysis—to test whether the combined intervention of ABM and CBT modifies AB toward alcohol—a GLM was used with AB‐RT as the response variable, group and time as explanatory variables, and age and initial AB‐RT value as covariates. As a second supplementary analysis, data from participants who relapsed during the intervention period were calculated using the Smirnov‐Grubbs test. Statistical analysis was conducted using Jamovi Version 2.0 software (the Jamovi project, 2021, https://www.jamovi.org.), with a statistical significance level of 5%.

### 2.9 Ethical Considerations

This study was approved by the Research Ethics Committee of Saitama Prefectural University (Approval No. 24709) and the Ethics Committee of Narimasu Kousei Hospital (Approval No. 236). The trial was registered with the University Hospital Medical Information Center (UMIN Trial ID: UMIN000040530).

## 3 Results

### 3.1 Participant Recruitment Results

The participants were recruited between December 2019 and August 2022. The participants received interventions sequentially starting 2 weeks after admission. There were 34 participants in this study. Of these, two were discharged early due to the COVID‐19 outbreak (COVID‐19 GENOMICS UK CONSORTIUM, 2020). Therefore, the data from 32 cases were analyzed. There were 16 participants in each of the intervention and control groups.

The basic information about the participants is presented in Table 1. The number of participants who experienced relapse between post‐intervention and discharge was zero and two in the intervention and control groups, respectively. No adverse events occurred due to the implementation of the intervention with the added ABM treatment.

**TABLE 1:** Demographic and clinical characteristics, pre‐ and post‐intervention data for male participants.

Variables	Pre			Post		
	Intervention	Control	*p*_welch's *t*_	Intervention	Control	*p*_welch's *t*_
Age	46 (14)	47 (11)	0.55	—	—	—
AUDIT	32 (9.0)	33 (6.8)	0.69	—	—	—
Hospitalization (day)	88 (4.6)	88 (6.5)	0.41	—	—	—
γ‐GTP (IU/L)	77 (121.0)	73.5 (83.0)	0.26	—	—	—
POMS_TMD	4.5 (12)	3.5 (13.5)	0.32	—	—	—
VAS_craving (mm)	14 (18.5)	9.5 (14.5)	0.82	5.5 (8.5)	1.5 (18.0)	0.34
ARRS_Total	42.5 (12.5)	41.5 (7.5)	0.91	40 (8.8)	41 (8.3)	1.00
AB‐RT (msec)	855.8 (172.8)	779.1 (231.0)	0.32	743.6 (188.8)	738.0 (185.4)	0.48

*Note:* This table represents the basic information of the participants, along with their ARRS Total, Craving, and AB‐RT scores before and after the intervention. The study included 32 participants. The response variables were ARRS Total, Craving, and AB‐RT scores. The explanatory variables included group (intervention vs. control) and time (intervention period). Covariates included age, initial ARRS Total, initial Craving, and initial AB‐RT scores. Each item represents the median (interquartile range). Intervention: *n* = 16, Control: *n* = 16.

Abbreviations: AB‐RT, attention bias reaction time; ARRS, Alcohol Relapse Risk Scale; AUDIT, Alcohol Use Disorders Identification Test; IQR, interquartile range; POMS_TMD, Profile of Mood States_Total Mood Disturbance; VAS, Visual Analog Scale.

### 3.2 Analysis of Primary Outcomes

A GLM was used to verify the effect of adding ABM treatment simultaneously to male patients with AUD who received a 6‐week CBT program, with ARRS Total as the objective variable. The model fit was *χ* ^2^/df = 15.46, AIC = 544.9, and coefficient of determination *R* ^2^ = 0.70. The coefficients of each parameter used in the GLM are listed in Table 2. Regarding ARRS Total, when analyzing using GLM with intervention group and control group (group) and intervention period (time) as explanatory variables, and age and initial ARRS Total value as covariates, there was no interaction between group and time (*z* = −0.16, *p* = 0.87). Additionally, there was no main effect of group (*z* = 0.15, *p* = 0.89). However, there was a main effect of time (*z* = −2.58, *p* = 0.01). The Bonferroni post hoc test on the ARRS before and after the intervention showed a significant difference between the pre‐ and post‐intervention periods (*z* = 2.57, *p* = 0.04).

**TABLE 2:** Parameter estimation of intervention effects of ARRS, craving, and AB‐RT by generalized linear models.

	Parameter	Estimate	SE	95% confidence interval		Exp (B)	*z*	*p*	Post hoc comparisons—time_course			
				Lower	Upper				Difference	SE	*z*	*p*_Bonferroni_
ARRS	(Intercept)	42.375	0.401	41.589	43.162	2.53^18^	105.597	< 0.001				
	Group	0.117	0.809	−1.468	1.703	1.124	0.145	0.885	−0.117	0.809	−0.145	0.885
	Time	−2.531	0.983	−4.458	−0.605	0.080	−2.575	0.012	2.531	0.983	2.575	0.035
	Group × time	−0.313	1.966	−4.166	3.541	0.732	−0.159	0.874	0.258	1.390	0.185	1.000
Craving	(Intercept)	13.865	1.305	11.308	16.421	1.05^6^	10.628	< 0.001				
	Group	4.783	2.625	−0.363	9.928	119.458	1.822	0.072	−4.78	2.63	−1.82	0.072
	Time	−2.250	3.195	−8.513	4.013	0.105	−0.704	0.483	2.25	3.2	0.704	1.000
	Group × time	5.000	6.391	−7.525	17.525	148.413	0.782	0.436	−5.429	4.53	−1.199	1.000
AB‐RT	(Intercept)	762.625	9.313	744.373	780.877	Inf	81.892	< 0.001				
	Group	4.872	19.046	−32.457	42.202	130.61	0.256	0.799	−4.87	19.0	−0.256	0.799
	Time	−63.944	22.811	−108.653	−19.235	1.70^‐28^	−2.803	0.006	63.9	22.8	2.8	0.019
	Group × time	−17.225	45.622	−106.643	72.193	3.31^‐08^	−0.378	0.707	−1.51	32.5	−0.046	1.000

*Note:* The response variable is the score of ARRS Total, Craving, and AB‐RT; the group representing the intervention group and control group; and time representing the intervention period are explanatory variables. The covariates are age, initial value of ARRS Total, initial value of Craving, AB‐RT. Input the initial RT value for each. *n* = 32 (Intervention = 16, Control = 16). Gaussian probability distribution is used.

Abbreviations: AB‐RT, attention bias reaction time; ARRS, Alcohol Relapse Risk Scale; SE, standard error.

When verifying the intervention effect of adding 6 weeks of ABM treatment to male patients with AUD who received CBT, using GLM with craving as the objective variable, the model fit was *χ* ^2^/df = 163.35, AIC = 771.3, *R* ^2^ = 0.53. The analysis with craving as the objective variable, intervention and non‐intervention groups (group) and intervention period (time) as explanatory variables, and age and initial craving value as covariates using the GLM, showed no interaction between group and time for craving (*z* = 0.78, *p* = 0.44). Compared to CBT alone, the intervention combining ABM and CBT showed no main effect on group (*z* = 1.82, *p* = 0.07), and there was no main effect of time on craving (*z* = −0.7, *p* = 0.48).

### 3.3 Supplementary Analysis

To verify the intervention effect of simultaneously adding ABM treatment to male patients with AUD who received a six‐week CBT program, the fit of GLM with AB‐RT as the objective variable was *χ* ^2^/df = 8325.5, AIC = 1148.7, *R* ^2^ = 0.56. Analyzing AB‐RT as the objective variable, intervention and control groups (group) and intervention period (time) as explanatory variables, and age and initial AB‐RT value as covariates using GLM, there was no interaction between group and time for AB‐RT (*z* = −0.38, *p* = 0.70). Additionally, there was no main effect of group (*z* = 0.26, *p* = 0.80). However, there was a main effect of time (*z* = −2.8, *p* = 0.01). The Bonferroni post hoc test on the AB‐RT before and after the intervention showed that it was significantly lower after the intervention (*z* = 2.80, *p*_Bonferroni_ = 0.02).

The number of participants who relapsed between the start of the intervention and discharge was zero and two in the intervention and control groups, respectively. We conducted the Smirnov‐Grubbs test on each survey item in the control group for these two individuals, and Relapser A showed a higher initial craving value of 49 mm (*t* = 2.27, *p* = 0.03) compared to those who maintained abstinence. Furthermore, Relapser B showed a higher initial ARRS Total score of 63 points (*T* = 2.62, *p* = 0.01) compared to those who maintained abstinence (Appendix S3).

## 4 Discussion

### 4.1 Intervention of CBT Combined With ABM

This study examined the effects of combining ABM with CBT in male patients with AUD. The hypothesis that adding ABM to a CBT program would reduce Alcohol Relapse Risk Scale (ARRS) scores more effectively than a placebo ABM was not supported. Similarly, the intervention did not show a significant difference in reducing cravings compared with the placebo, indicating that the combination of ABM and CBT did not outperform CBT alone in this context.

### 4.2 Reasons Why ABM Did Not Affect Relapse Risk in Patients With AUD

Several studies have reported that ABM alone does not significantly reduce cravings, a primary symptom of AUD [43, 44].

A systematic review of the efficacy of ABM in substance use disorders found no significant effects on symptoms associated with changes in attentional bias [45]. Additionally, studies comparing relapse rates in groups receiving CBT versus usual treatment found that CBT significantly reduced relapse rates within 1 year [46]. These findings suggest that the observed reduction in relapse risk in both groups was due to CBT rather than ABM.

### 4.3 Reasons Why ABM Did Not Affect Craving in Patients With AUD

In this study, ABM did not significantly impact craving scores in patients with AUD. Although there is generally a correlation between attentional bias (AB) toward alcohol and cravings [29], this relationship may be influenced by metacognitive factors [47]. Field et al. [48] reported that the association between AB and alcohol cravings is weaker than that for other substances such as caffeine or cannabis. Moreover, patients with AUD under treatment often avoid alcohol‐related visual stimuli more than neutral stimuli [49, 50, 51], indicating a stage where they are actively trying to mitigate cravings by modifying their AB.

Neuroimaging studies indicate that psychosocial stress impairs selective attention control and weakens the functional connectivity within the frontoparietal network [52]. Under psychological stress, the ability of the prefrontal cortex to distribute attention diminishes, while the amygdala and striatum become more active, driving habitual and impulsive emotional responses [53, 54].

In men, the strength of cravings correlates significantly with striatal activation [55]. Chronic or prolonged exposure to stress can overstimulate the prefrontal‐limbic‐striatal circuits, exacerbate psychological distress, and increase the risk of craving and seeking alcohol. Therefore, interventions targeting prefrontal cortex function or impulse control may be more suitable than ABM for patients with AUD and high cravings.

It is also possible that cravings and AB are controlled by different mechanisms. Thus, a detailed evaluation of patients' AB toward alcohol is necessary, and interventions combining CBT with ABM should be tailored to individual cognitive characteristics. Considering the sequence of attention, cognition, and behavior [56, 57], cravings might diminish after effective avoidance of AB is established. While previous research has indicated that more than 800 trials are needed for ABM to be effective [31, 32], this study's sessions—which exceeded 800 trials—may not have accounted for the variability in initial craving levels among participants. Consequently, the lack of an effect of ABM on craving observed in this study could be attributed to these factors. Future research should consider individual patient characteristics to optimize ABM efficacy.

### 4.4 Challenges in ABM Used in This Study

The dot‐probe task used in this study required participants to identify alcoholic and non‐alcoholic images with the aim of training them to divert their attention from alcoholic stimuli. Effective training should consider the complexity of real‐world environmental factors that influence alcohol use [58]. In real‐life settings, external factors such as advertisements, peer pressure, and environmental stressors can make it difficult for individuals to resist alcohol [59]. The participants in this study were inpatients who were isolated from societal stressors, which may have impacted the results. Cheng et al. [60] reported that stress was correlated with alcohol cravings in patients motivated for treatment. The participants were exposed to fewer alcohol‐related stimuli in the inpatient setting, which may have affected the study outcomes. AB is more pronounced in familiar environments where participants are accustomed to consuming alcohol [58].

Another study has shown that cue exposure therapy using Virtual Reality (VR) can enhance treatment by simulating real‐life drinking scenarios [59]. Therefore, future studies should incorporate both images of alcohol consumption and situational cues to measure AB accurately. This suggests that ABM is more effective in settings related to alcohol use, such as home environments, than in clinical or laboratory environments unrelated to alcohol consumption. Thus, the environmental limitations of this study may underestimate the impact of ABM on realized actual drinking behavior and relapse risk [61].

Previous studies have shown the placebo effects of ABM [62, 63]. The supplementary analysis indicated that both ABM and placebo reduced AB‐RT, suggesting some placebo effect. Participants were unaware of their group allocation, which could have influenced their adherence to the training. Research on sports has indicated that participants' beliefs in the intervention group can enhance adherence [64]. Adjusting trial ratios to include alcohol and non‐alcohol images in the placebo training may have influenced the results, demonstrating a placebo effect.

While our study focused on basic attentional processes using non‐alcoholic beverages as neutral stimuli, future research should consider incorporating more functionally relevant alternative stimuli. Attention bias modification could potentially be enhanced by using images that represent adaptive coping behaviors and positive lifestyle choices, such as:

1. Engaging in recreational activities that provide alternative sources of pleasure and reward.
2. Positive social interactions without alcohol that fulfill social connection needs.
3. Stress management activities that address underlying triggers for alcohol use.
4. Healthy lifestyle activities that promote overall well‐being.

This approach would strengthen the bridge between the automatic attentional processes targeted by ABM and the functional behavioral alternatives emphasized in CBT. By incorporating stimuli that directly address the psychological functions served by alcohol use, future interventions might achieve stronger therapeutic effects through the integration of bottom‐up attentional modification and top‐down behavioral strategies.

### 4.5 Effect of Interventions on Relapse Risk in Inpatient Male Patients With AUD

While there was no significant difference in relapse risk reduction between the intervention and control groups, a reduction was observed across all participants after 6 weeks. Previous research reported ARRS scores of 45.9 (SD = 0.32) for those who relapsed within a month and 41.3 (SD = 0.30) for those who maintained abstinence [36]. In this study, the initial ARRS total score was 43.8, decreasing to 41.3 after 6 weeks. This suggests that both ABM and placebo interventions reduced relapse risk to a level comparable to those maintaining 1 month of abstinence.

### 4.6 Limitations

This study had a selection bias as participants voluntarily chose to join the CBT program, indicating high motivation for treatment and abstinence. Nevertheless, motivational levels varied among inpatients. Diclemente, Bellino, and Neavins [65] found that motivation significantly impacted behavioral changes in patients with AUD. Furthermore, Witkiewitz and Hartzler [66] reported that interventions enhancing motivation in individuals with AUD reduced drinking frequency. This study did not assess the participants' motivation for abstinence, which could have influenced the results. For accurate ABM effects, the assessment and consideration of patient motivation for treatment and abstinence are crucial. Participants were self‐selected inpatients, resulting in a selection bias in the baseline data for relapse risk and craving. Future studies should evaluate motivation levels and compare them with groups that do not opt for CBT.

The ARRS data comparison revealed this study's participants had a lower relapse risk, possibly reducing the impact of the intervention. However, patients who relapsed in this study had higher baseline relapse risk scores. Psychological factors related to positive expectations from alcohol play a role in relapse [67]. This suggests that individuals with a higher relapse risk may benefit more from ABM interventions tailored to reduce cravings and AB.

The study was conducted in an inpatient setting, which might have limited the effectiveness of ABM due to a lack of real‐world alcohol‐related stressors. Future research should consider multicenter studies, including both open‐ and closed‐ward environments, to verify these findings. This study focused on male inpatients who volunteered for a CBT program, thus limiting its generalizability. In addition, environmental factors and treatment motivations must be considered in future studies to ensure a comprehensive evaluation.

The COVID‐19 pandemic potentially influenced our study in several ways. First, pandemic‐related stress may have affected participants' psychological status and coping mechanisms. Second, hospital protocols were modified to ensure safety, potentially affecting treatment delivery and participant interactions. While two participants were discharged early due to COVID‐19 protocols, we maintained consistent study procedures throughout the trial. Future research should consider how such extraordinary circumstances might impact treatment outcomes and protocol implementation.

## 5 Conclusion

This study investigated the impact of ABM combined with CBT on relapse risk and cravings in male inpatients with AUD. The findings indicated that the ABM and placebo interventions effectively reduced relapse risk, but there was no significant difference between the two groups. Additionally, neither intervention significantly altered the patients' cravings for alcohol. These results suggest that a 6‐week CBT program is effective in reducing relapse risk in male inpatients with AUD. However, further research is needed to evaluate the specific conditions under which ABM may influence cravings, including detailed assessments of attentional bias and individualized training protocols.

## Author Contributions

**Yoshifumi Amano:** conceptualization, methodology, data curation, writing – original draft preparation. **Toyohiro Hamaguchi:** data curation, project administration, supervision. **Kohei Koizumi:** writing – reviewing and editing. **Hirokazu Takizawa:** writing – reviewing and editing. **Shota Tasaka:** writing – reviewing and editing.

## Ethics Statement

This study was approved by the Research Ethics Committee of Saitama Prefectural University (Approval No. 24709) and the Ethics Committee of Narimasu Kousei Hospital (Approval No. 236). The trial was registered with the University Hospital Medical Information Center (UMIN Trial ID: UMIN000040530).

## Consent

Informed consent was obtained from all individual participants included in the study.

## Conflicts of Interest

The authors declare no conflicts of interest.

## Supporting information


Appendix S1

Appendix S2

Appendix S3

## Data Availability

The complete raw dataset that supports the findings of this study is publicly available in the Mendeley Data repository (Hamaguchi [68]).
