# Supplementary material for: Effectiveness of Attentional Bias Modification Combined With Cognitive Behavioral Therapy in Reducing Relapse Risk and Cravings in Male Patients With Alcohol Use Disorder: A Quasi‐Randomized Controlled Trial
Source: Neuropsychopharmacol Rep. 2025 Feb 5;45(1):e70002. doi: 10.1002/npr2.70002 (PMC11795173; doi:10.1002/npr2.70002)
Supplement: Supplementary file 1 — Appendix S1 [file NPR2-45-e70002-s002.pdf]

Appendix I. Coping Skills Training Workbook Used in the Intervention. The workbook consisted of six sessions.

The program sessions lasted 80 minutes each. The program was conducted by occupational therapists, certified psychologists, and nurses. The number of participants was approximately 10 per session. The procedure was detailed in a textbook, and care was taken to minimize differences in interventions between facilitators.

Please check if this should be Appendix Ⅱ. Contents of Coping Skills Training for Alcohol Dependence (to match the wording in the Excel file).

CST

Coping skills training

Program to think about how to stop drinking alcohol

Name：____________________

Unauthorized use and reproduction of this text is prohibited.

Contents

・Welcome to CST・・・・・・・・・・・・・・・・・・・・・・・・・・・・・・・・・・・

・ Part 1: What is alcohol for you? ・・・・・・・・・・・・・・・・・・・・・・

・ Part 2: How to deal with cravings and triggers ・・・・・・・・・

・ Part 3: Your own dangerous patterns and solutions ・・・・・・

・ Part 4: Changing your mindset towards alcohol ・・・・・・・・・

・ Part 5: Let's practice refusing alcohol ・・・・・・・・・・・・・・・・・

・ Part 6: Think about what to do if you start drinking again ・・・

Briefly explain the contents according to the table of contents

Be sure to read the participation agreement. Inform participants that they will have limited time to speak, so they will have to share speaking time.

Welcome to CST

This program is for people who want to quit drinking alcohol. You can talk about how to quit drinking with people who have similar experiences and worries, and think about your future treatment and life. Our specialist staff will also provide you with knowledge and information that will be useful in the future.

Stamp

1	2	3	4	5	6

Promise to participate

①Please listen to the other members until the end.
②Please refrain from criticizing or attacking the opinions of other members.
③Let's talk about what we think and feel.
④Anything discussed during the program should not be discussed outside the program.
⑤You may leave at any time. Please participate at your own pace.
⑥ The program includes homework, which you should complete before your next participation.

Part 1.
What is alcohol for you?

Goals of today's session

○Learn about coping skills
○Verbalize your feelings about alcohol treatment.
○Verbalize your feelings about alcohol.

What are coping skills?

Translated into Japanese, this means "coping skills." Coping skills are acquired through practice. Once you have acquired coping skills, you will be able to stay away from alcohol in dangerous situations in an appropriate way.Over the course of six sessions, you will become aware of dangerous situations and acquire new coping skills while receiving advice.

Read the purpose.
Explain coping skills.

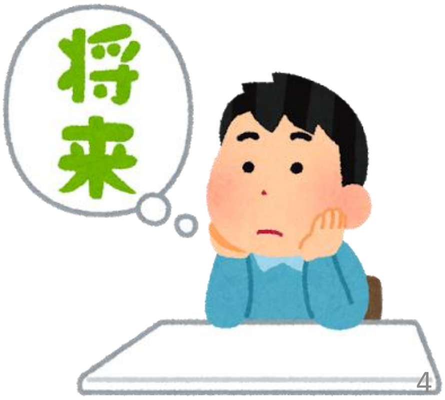

Q1

<u>How do you feel about wanting to drink alcohol right now and how confident are you that you can quit? Please underline the answers below.</u>

① I want to drink alcohol. ⇔ I want to stop drinking.

0 ←→ 100

I never want to stop
I want to drink somehow

I want to completely stop drinking alcohol

② Strength of will to stop drinking and confidence to stop drinking

0 ←→ 100

If there's alcohol in front of me, I'll drink it.

Even if there is alcohol in front of me, I won't drink it.

Listen to the opinions of a few people Whether to listen to everyone depends on the number of participants. Let them know that there is no problem even if they are not 100% determined to quit. Where does caution and confidence come from? People who have concrete ideas about their future sobriety may be more anxious.

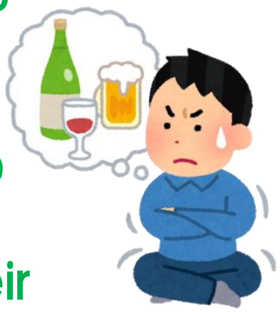

Q2

Have you ever tried to quit drinking? How did it go?

What I tried.	Result.

Write in about 2-3 minutes.
Ask several people.
Key point: It is difficult to maintain abstinence from alcohol. Rules and methods that you come up with on your own tend to change to suit your own convenience.→ It is important to make use of rules that you have decided on in consultation with others, and methods that other people have used to successfully abstain from alcohol.

Advantages/Disadvantages of Alcohol

What are the advantages (good points) and disadvantages (bad points) of drinking alcohol? Have you ever thought about the advantages and disadvantages of drinking alcohol? This may give you an opportunity to think about your life from now on, so let‘s take a look back at how you have drunk alcohol up until now. Fill in the table on the next page while remembering how you have drunk up until now.

What are the advantages and disadvantages of drinking alcohol to you?

Advantages of drinking alcohol	Disadvantages of drinking alcohol

Advantages of quitting alcohol	Disadvantages of quitting drinking
例）家族との関係が良くなる	例）眠れなくなる

The benefits of drinking tend to have momentary or transient effects, while the benefits of quitting tend to have long-term effects. When you want to drink, you tend to remember the good things you did when you drank, and that's the reason you end up drinking. Focus on the disadvantages of drinking and the advantages of not drinking.

Part 2: How to deal with cravings and triggers

Goals of today's session

- ○ Learn about cravings and triggers
- ○ Reflect on your triggers.
- ○ Think about how to deal with cravings.

What is craving?

The physiological urge to drink alcohol.

Cravings are your brain's way of telling you to drink. They can occur even if you've resolved to quit or have been sober for years.

What is a trigger?

It refers to the people, places, things, situations, and emotions that trigger the desire to drink.

To avoid the desire to drink ･･･

1. Know your triggers.
2. 2. Avoid your triggers.

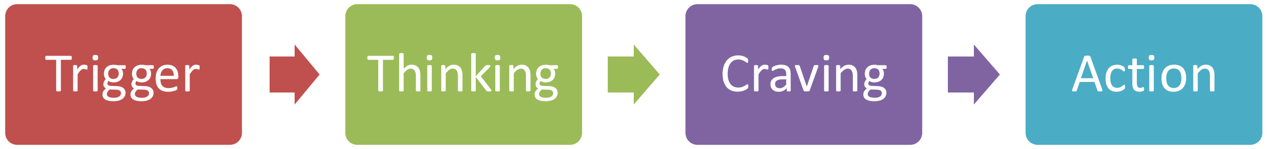

What is your trigger?

People / Places / Situations	Emotions
☐Friends ☐Drinking buddies<br>☐Family ☐Other<br>☐Colleagues/bosses<br>( )<br><br>☐Home ☐Convenience store<br>☐ Friend's house<br>☐ On the way home from work<br>☐ Weddings, funerals, etc.<br>☐Karaoke ☐Other<br>( )<br><br>☐ When I'm home alone<br>☐ When I have money<br>☐ The day before a day off or on a day off<br>☐ When I can't sleep ☐ At mealtimes<br>☐ When I'm bored ☐ When I'm cooking<br>☐ When it's cold or hot ☐ After work<br>☐ After a self-help meeting<br>☐ Other times<br>( )	☐Irritated<br>☐Depressed<br>☐Anxious<br>☐Tired<br>☐Sad<br>☐Lonely<br>☐Feeling left out<br>☐Happy<br>☐Relaxed<br>☐Headache<br>☐Hungry<br>☐Other<br>( )<br>☐Other<br>( )

When you get a craving for alcohol・・・

<u>The cravings disappear after a certain amount of time ！</u>

When you find yourself wanting a drink, it can be reassuring to learn ways to get through the time until the urge passes.

How to Deal with Cravings

- Take action to distract yourself

- Change the way you think about alcohol

  (Think about the downsides of drinking alcohol .)

Generate various ideas for how to deal with the situation. Create a matrix of "Alone," "multiple people," "outdoors," and "indoors," and write down ways to do the situation within 15 minutes.

Solution: Something that can be done within 15 minutes

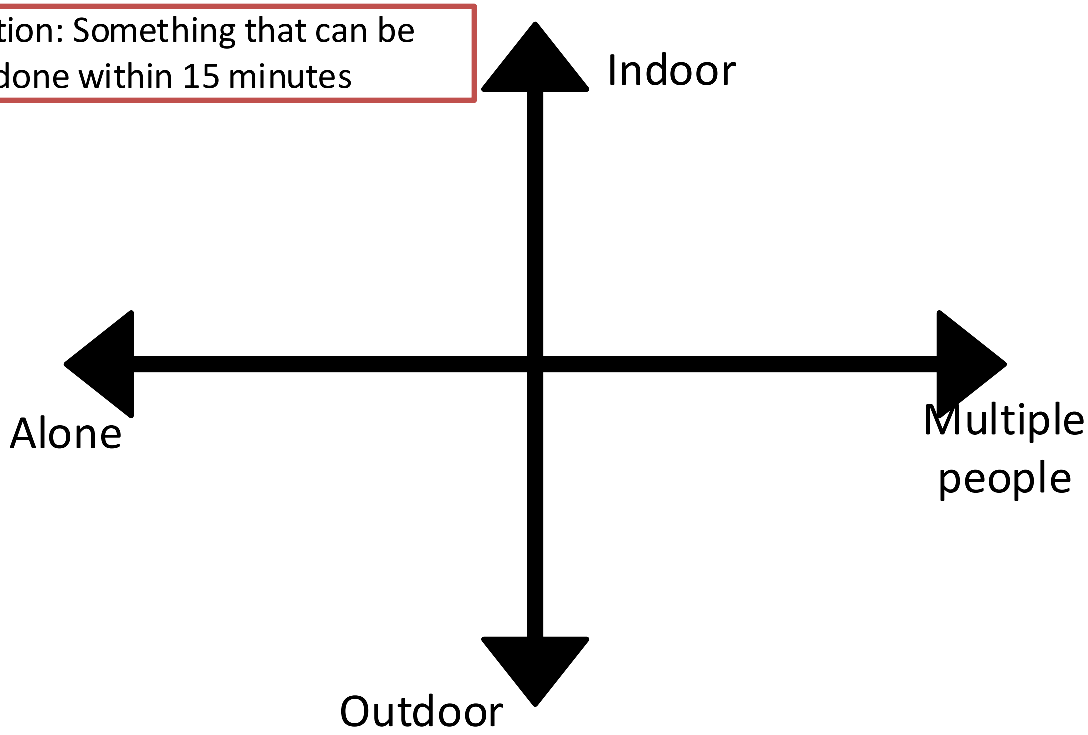

Ride the wave of your desire to drink

Desires are like waves, sometimes strong and sometimes weak.
They eventually disappear and do not linger for long.
Here, we will introduce a method, through mindfulness meditation, of surrendering yourself to the waves of desire and letting them pass.

①Sit in a chair in a comfortable position.

Place your feet on the ground and your hands in a comfortable position.

②Take a few deep breaths.

③Bring your attention inward.(Where is the desire? Mouth, nose, head?)

(What sensations are you feeling? Hot, cold, fatigue, numbness, dryness?)

④Repeat focusing on each part of your body.

Part 3: Your own dangerous patterns and solutions

Goals of today's session
○Learn about dangerous situations.
○Learn the skills to not drink alcohol.

It's difficult to continue a life without drinking in a society where alcohol is so prevalent.

Work drinking parties, ceremonial occasions, social gatherings...You can easily get alcohol by stopping by a convenience store.

If you travel, alcohol is also sold on board transport.

In order to live in such an environment, it's important to find ways to protect yourself.

Dangerous situations that make you want to drink alcohol.
Draw a vertical line around the strength of your desire to drink.

▪ **Example: When you see a beer commercial**

No Alcohol	←——|——→	I want to drink alcohol

▪

No Alcohol	←————→	I want to drink alcohol

▪

No Alcohol	←————→	I want to drink alcohol

▪

No Alcohol	←————→	I want to drink alcohol

▪

No Alcohol	←————→	I want to drink alcohol

▪

No Alcohol	←————→	I want to drink alcohol

▪

No Alcohol	←————→	I want to drink alcohol

▪

No Alcohol	←————→	I want to drink alcohol

▪

No Alcohol	←————→	I want to drink alcohol

▪

No Alcohol	←————→	I want to drink alcohol

Homework: Ask participants to describe situations in which each desire was particularly strong and weak.

Q1

If you encounter a dangerous place or situation, what will you do? (Think about how to deal with the situation on the previous page.)

Homework
Change your coping behavior depending on the strength of your desire to drink.
The stronger the desire, the stronger the coping behavior.
Rather than throwing all your energy into dealing with every desire to drink, focus on the key points and deal with them.

Q2

Consider good and bad ways to deal with the situation if you start drinking again after being discharged from hospital.

Good⇒⇒

Homework
Good : Asking for help. Being honest.
Bad : Hiding the fact that you drank. Trying to manage on your own.

Bad⇒⇒

Q3

① Are there any aspects of your daily routine during hospital stay that would help you quit drinking after you are discharged?

Homework
Implementation means what kind of ideas you can come up with to apply it to your own life. How to make it more practical.
Examples: 1. Exercise 2. Get up early in the morning and make time to run.

② What kind of ideas can you come up with to incorporate this into your life?

Q4

What are some good/bad things about self-help groups?

Good⇒⇒

Bad⇒⇒

Homework
Self-help groups have a function called "role modeling."
A major benefit of self-help groups is that you can meet many people who have recovered from illness.

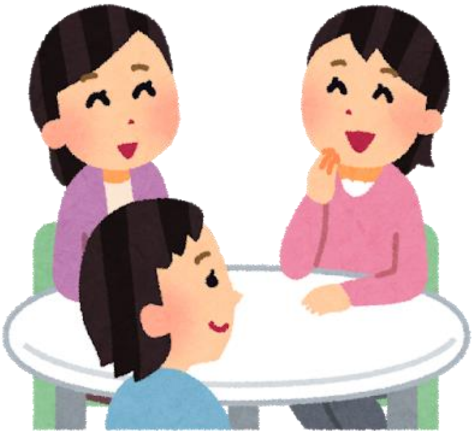

When you find yourself in a dangerous place or situation, or when you hear dangerous whispers from within yourself, the only thing you can do to counteract them is to take actions that will help you recover.

For example, go to an outpatient clinic. Go to a self-help group. Read books about recovery. Talk to friends who have stopped drinking. Exercise. Go to day care, etc.

Imagine your life after you are discharged from hospital, reflect on dangerous situations you have been in, and learn how to deal with them. Listen to the stories of others and widen the range of actions you can take to recover.

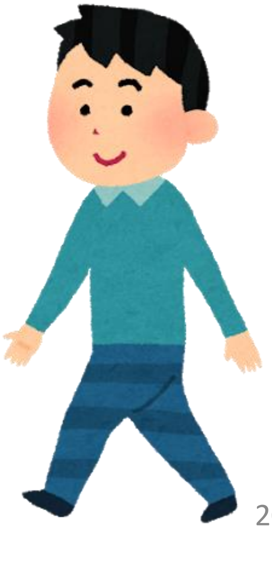

Part 4: Changing your perception of alcohol

Goals of today's session

○ Understand the things and situations that put you at high risk for relapse

○ Learn about sabotaging thoughts.

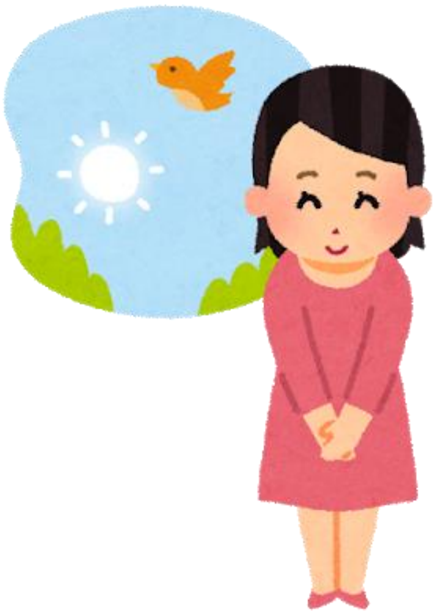

High risk of relapse

①　When you want to escape from unpleasant things

Failure, rejection, disappointment, injury, humiliation, embarrassment, dissatisfaction, sadness…When you find yourself in such a situation, seeking "security" in alcohol will lead to relapse.

②When you want to relax

The desire to relax is normal, but it can be dangerous when you are in such a hurry to relax that alcohol becomes the means.

③ Relief of tension

Similar to relaxation, people who feel embarrassed or uncomfortable in public may need alcohol as a lubricant to make them feel more comfortable and less awkward.

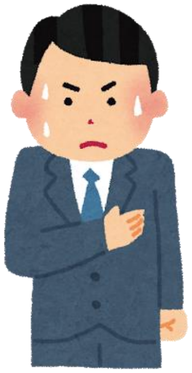

④ Improved self-esteem

When I'm dissatisfied with myself, when I feel inferior to others, when I feel unattractive, I think about alcohol.

⑤love affair

Love becomes a thrill. When I'm in love, I think I need alcohol to make it more fun.

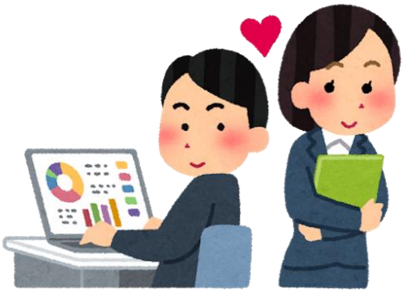

⑥ Feelings of despair

When you lose sight of your goals for the future, you also lose hope. Is it necessary to think about or try alcohol at this time? At times like these, your wariness of alcohol tends to fade.

⑦ Trying out

At some point, you may start to think, ″Am I really unable to drink anymore?″ Alcohol creates this kind of expectation for you.

Automatic thoughts
# – Cognitive distortions regarding alcohol –

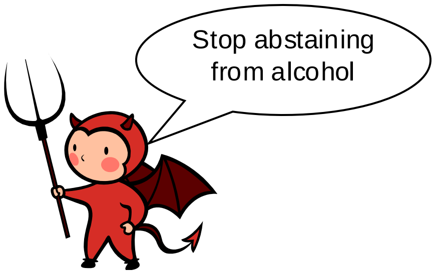

- Thoughts that interfere with sobriety:

These thoughts unconsciously pop into your mind when you are in a certain situation (when you remember an event).

1. The idea of allowing to drink
2. Thoughts of harming yourself
3. Ignoring advice
4. Thoughts that increase stress

Types of automatic thoughts

- **People who fail to quit drinking have a characteristic flaw in the logic of their thinking.**

- All-or-nothing thinking A state in which you think there are only two types of situations: "good" and "bad."
- Jump to conclusions

  Jumping to conclusions without any valid evidence
- Negative future predictions

  I have only a pessimistic view of the future
- Positive future predictions

  I'm optimistic about the future
- Emotional meaning

  Making decisions based on emotions
- Underestimating the good

  Underestimating the good things and things that went well
- Labeling

  Labeling yourself, other people, or things without any proof, and assuming that "that's just the way they are.“
- Misreading other people's feelings

  Believing what others around you think based on your own assumptions
- Deceiving yourself

  Thinking that everything is your responsibility
- Useless rules

  Bound by negative rules you've made up
- Misguided

  Assuming that something is the cause when in fact it has nothing to do with you
- Exaggerated thinking

  Thinking that you should do something

Types of automatic thoughts

• For example ••

- All or nothing thinking

No matter where you go to a self-help group, it's always the same story

- Rushing to a conclusion

Staff won't talk to you“

They don't care about me“

- Negative future predictions

I'll just drink and fail again

Even if I'm honest, I'll get scolded and it'll be over

- Positive future predictions

No one will find out if I drink

Drinking will make things better

- Emotional meaning

It's annoying and upsetting because it's his fault

- Underestimating the good things

Going a day without drinking is no big deal

- Labeling

Abstinence is very hard

- I have no self-control

Misreading the other person's feelings

- The other person must want to drink with me

Deceiving myself

It's all my fault that things have turned out this way

- Useless rules

I don't get drunk on beer

Drinking at weddings is inevitable

- Misguided

If you can't drink, people will think you can't work

- Overly thoughtful

Abstinence is so hard that people around you should be more considerate

Column Method – Table-Based Intervention

Example

Situation	At home, she discusses her future plans with her family. They get into an argument and the family leaves, leaving her alone. She is on the road to recovery, including attending self-help groups.
Thoughts at the time	I'm trying so hard for myself and my family, why don't they understand? Should I just stop trying and drink?
Classification	All-or-nothing thinking<br>Rushing to conclusions<br>Negative predictions<br>Positive predictions<br>Underestimating good things<br>Emotional meanings<br>Labeling Misreading other people's feelings<br>Self-deceiving thinking Useless rules<br>Misguided Exaggerated thinking
Revised thoughts	I can have this discussion because I'm sober. I should think about the other person's feelings more. My family is also thinking about it seriously. If I drink, I won't be able to stay calm and my efforts will be wasted.
Expected changes	You can deal with the situation honestly. It will strengthen family ties. It may bring about positive changes for your family and yourself. It may help you avoid arguing next time.

Column Method - Table-Based Intervention

Work to change your mindset

Situation	
Thoughts at the time	
Classification	All-or-nothing thinking<br>Rushing to conclusions<br>Negative predictions<br>Positive predictions<br>Underestimating good things
Revised thoughts	
Expected changes	

•If the scene is long, it is good to focus on ”what prompted them to drink.“

•It is easier if the scene is based on a past experience, but fictional scenes are fine too.

•After categorizing, ask other people for their opinions and broaden your way of thinking.

•It is good to connect the revised thoughts to thoughts that are ”positive” to you.By categorizing, you can analyze your own thinking habits.

•If you think alone, you can only make changes within your own thinking patterns. It is important to incorporate other people's thinking and broaden your way of thinking.

Part 5: Let's practice refusing alcohol

Goals of today's session
〇Learn how to respond when offered alcohol
〇Experience persuasive ways to refuse

What situations, people, and places would it be difficult for you to refuse alcohol if it was offered to you?

Anticipate situations you may encounter after being discharged from hospital and think about how to deal with them.

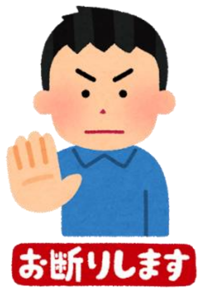

Q1

When is it difficult for you to refuse alcohol?

Q2

Who is the person you find it hard to refuse when they offer you a drink?
Name them specifically.

Homework
Analyze the situation and the person separately.
What kind of situation would be difficult to refuse?
What kind of person would be difficult to refuse?

Q3

From Q1 and 2, write down the specific situations, people, and places that are most difficult to refuse, and think about how to deal with them.

	situation, person, and location	Workaround
1		
2		
3		
4		
5		

Tips on how to communicate

- **Speak with focus**

  Be specific and state one wish.

  It is best to speak in short, concise sentences.

- **Return to what you want to say**

  Stay calm and get back to what you want to say. This will help you avoid arguments and going off on tangents.

- **Expressing your feelings**

  Express your feelings in words and communicate them to the other person.

- **Use body language when speaking**

  Pay attention to your attitude, facial expressions, and tone of voice. By matching your words with your facial expressions and attitude, you can clearly communicate what you want to say.

- **Try to understand the other person's feelings**

  Let's value ourselves and others. Adding words that help us understand others will make communication easier.

How to decline an invitation to go drinking

＊How to decline
・I have a prior engagement...
・My doctor has told me not to drink alcohol
・Unfortunately, I have an appointment that I cannot miss
・I made a promise with my child
・My liver has been damaged
・I am abstaining from alcohol due to diabetes
・Because I cannot drink alcohol (I always make it clear that I am a lightweight)
・I joined an AA
・I cannot drink alcohol, so I will excuse myself from the drinking party. I will join if the restaurant has good food.
・My wife is in bed, so I have to look after my children

＊How to deal with it
・Ask the organizer to prepare soft drinks
・Tell the organizer that you cannot drink.
・Bring juice or tea with you
・Don't go on an empty stomach
・Leave after the greetings and toast
・Sit next to someone who doesn't drink
・Don't attend family gatherings (protect yourself even if it means going out of your way to do things)
・Take anti-alcohol medication
・Contact your self-help group members in advance to avoid feeling lonely

Role play

A learning method in which people imagine a realistic situation, play roles, and use simulated experiences to prepare them to respond appropriately when something actually happens.

★Method

① Practice refusing alcohol by choosing the situation and person you think is the hardest to refuse from those given in Q3.

Actor: The person refusing alcohol. The person playing your role.

You refuse alcohol.

Briefly explain the situation in which you would refuse alcohol.

Please provide specific information so that your partner can play the role.

Partner: Play the role of the person offering alcohol.

Observer: Observe how the actor refuses alcohol.

Pay attention not only to the words but also to facial expressions and attitudes.

② After the actor has actually acted out the situation, the partner/observer will give feedback. The actor should tell us what they thought.

In the feedback, the actor should tell the other person what they liked about what they saw. If there is one thing that could be improved, tell them just one thing.

The important thing is not to criticize or deny.

③The actor who received the feedback will perform the situation again, taking the advice and tips on how to communicate into consideration. Once it's finished, give feedback again.

Part 6: Prepare for the worst and think about how to deal with relapses

Goals of today's session

〇 Consider appropriate measures to take if you start drinking again

〇 Create a schedule for staying safe after being discharged from hospital

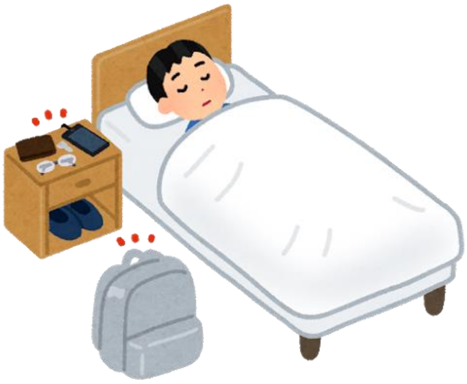

Planning for risky situations: Cravings

What to do in situations where you feel the urge to drink:

1. Leave the situation.
2. The overwhelming desire to drink will not last forever. Remember that it will gradually disappear, and set aside the choice to drink for 15 minutes.
3. Question the choice to "drink." For example, "Do I really need to drink now?", "Maybe I don't need to drink," or "Will drinking make my condition worse?“
4. Think about something completely unrelated to drinking
5. Remember a time when you were able to get through the urge to drink
6. Contact your emergency list (trusted people, places)

Emergency List

Name	Number
1、	
2、	
3、	
4、	
5、	
6、	

Make a list of people they can consult in an emergency. However, a golden rule is to consult before starting to take medicine. It is ideal to choose friends who understand the illness. Also, explain that AA has a sponsorship system. Participants are asked why they chose who they were.

Planning for risky situations: Relapse

The first sip is an emergency. It takes a lot of effort to maintain stable abstinence.

What you can do if you try to quit but end up taking a sip for some reason.

1. Get rid of all alcohol.
2. Don't let guilt or self-criticism overwhelm you and take action to stop yourself from continuing to drink.
3. Get help.
4. Reflect with a professional (your doctor or a caregiver) on what triggered the first sip and your reaction to it. By exploring the effects you secretly expect from alcohol and the things you think alcohol will change, you can prepare to prevent the same thing from happening again.

Stopping the first sip is the shortcut to recovery. Once you have taken a sip, it will be a detour, but do what you can to prevent yourself from going back to your old state.

	Mon	Tue	Wed	Thu	Fri	Sat	Sun
AM							
PM							
AA							

Homework

Have them write down the schedule with the image of [starting from the day they are discharged from the hospital] in mind.

-Balance with days

Schedule your day

5:00		12:00		19:00	
5:30		12:30		19:30	
6:00		13:00		20:00	
6:30		13:30		20:30	
7:00		14:00		21:00	
7:30		14:30		21:30	
8:00		15:00		22:00	
8:30		15:30		22:30	
9:00		16:00		23:00	
9:30		16:30		23:30	
10:00		17:00			
10:30		17:30			
11:00		18:00			
11:30		18:30			

Homework

Look back on the time when you used to drink, imagine a dangerous day for you, and create a "schedule to not drink."

To establish a rhythm in your life, it is good to be able to fix your wake-up and bedtimes.

CST - Completed all 6 sessions. Thank you for your hard work.

Have you acquired a new way of thinking about alcohol and how to deal with dangerous situations?

Your life after you are discharged from hospital is the real beginning.

Use what you have learned during your stay in hospital to live a better life.

Our center is always available to contact you. If a dangerous situation arises, we hope that you will pick up the phone instead of turning to alcohol.
