# Supplementary material for: Effectiveness of Attentional Bias Modification Combined With Cognitive Behavioral Therapy in Reducing Relapse Risk and Cravings in Male Patients With Alcohol Use Disorder: A Quasi‐Randomized Controlled Trial
Source: Neuropsychopharmacol Rep. 2025 Feb 5;45(1):e70002. doi: 10.1002/npr2.70002 (PMC11795173; doi:10.1002/npr2.70002)
Supplement: Supplementary file 2 — Appendix S2 [file NPR2-45-e70002-s003.docx]

| Appendix Ⅱ. Contents of Coping Skills Training for Alcohol Dependence | | |
| --- | --- | --- |
| **Session** | **Title** | **Content** |
| 1 | What Does Alcohol Mean to You | Discuss the motivations for sobriety and the pros and cons of drinking. |
| 2 | Coping with Cravings and Triggers | Identify situations where cravings are felt and share strategies for coping with these cravings. |
| 3 | Recognizing and Solving Your Risky Patterns | Reflect on situations that induce cravings and the intensity of these cravings. Consider how to generalize inpatient experiences to life after discharge. |
| 4 | Changing Your Thoughts About Alcohol | Address thoughts that lead to deviations from sobriety using the column method for cognitive restructuring. |
| 5 | Practicing Ways to Refuse Alcohol | Practice ways to refuse alcohol when offered by acquaintances. |
| 6 | Preparing for Relapse Management | Clarify the contact points for support in case of relapse and plan the first week's schedule after discharge. |
| The program sessions lasted 80 minutes each. The program was conducted by occupational therapists, certified psychologists, and nurses. The number of participants was approximately 10 per session. The procedure was detailed in a textbook, and care was taken to minimize differences in interventions between facilitators. | | |
