# Supplementary material for: Effectiveness of Attentional Bias Modification Combined With Cognitive Behavioral Therapy in Reducing Relapse Risk and Cravings in Male Patients With Alcohol Use Disorder: A Quasi‐Randomized Controlled Trial
Source: Neuropsychopharmacol Rep. 2025 Feb 5;45(1):e70002. doi: 10.1002/npr2.70002 (PMC11795173; doi:10.1002/npr2.70002)
Supplement: Supplementary file 3 — Appendix S3 [file NPR2-45-e70002-s001.docx]

| Appendix Ⅲ. Calculation of Outliers among Participants who Relapsed Using the Smirnov-Grubbs Test. | | | | | | | | |  |  |  |
| --- | --- | --- | --- | --- | --- | --- | --- | --- | --- | --- | --- |
|  | All Participant | 95% confidence Interval | | Participant A | Smirnov-Grubbs | | Participant B | Smirnov-Grubbs | |  | |
| Variables | Median(IQR) | Lower | Upper | raw score | T | p | raw score | T | p |  |  |
| age | 46.5 (12.0) | 41.96 | 48.5 | 41 | 0.47 | 0.65 | 54 | 0.95 | 0.35 |  |  |
| AUDIT | 32 (8.25) | 27.98 | 32.4 | 30 | 0.03 | 0.98 | 36 | 0.95 | 0.35 |  |  |
| POMS_TMD | 3.5 (12.25) | 2.82 | 12.5 | 5 | 0.2 | 0.84 | 3 | 0.35 | 0.73 |  |  |
| Craving (mm) | 11 (19.25) | 8.67 | 19.7 | 49 | 2.27 | 0.03 | 22 | 0.51 | 0.61 |  |  |
| ARRS_Total | 42 (9.75) | 41.14 | 46.4 | 47 | 0.44 | 0.66 | 63 | 2.62 | 0.01 |  |  |
| AB-RT (msec) | 828.95 (203.77) | 744.87 | 844 | 761.8 | 0.24 | 0.81 | 853.5 | 0.43 | 0.67 |  |  |
| This table shows the values of age, AUDIT, POMS TMD, Craving, ARRS Total, and AB-RT for all participants. For those who relapsed during the intervention period, outliers were tested using the Smirnov-Grubbs method. n = 32.  AUDIT: Alcohol Use Disorders Identification Test. POMS_TMD: Profile of Mood States_Total Mood Disturbance.  ARRS: Alcohol Relapse Risk Scale. AB-RT: Attention Bias Reaction Time. | | | | | | | | | |  |  |
|  |  |  |  |  |  |  |  |  |  |  |  |
|  |  |  |  |  |  |  |  |  |  |  |  |

| Descriptives | |  |  |  |  |  |  |  |  |  |  |  |  |  |
| --- | --- | --- | --- | --- | --- | --- | --- | --- | --- | --- | --- | --- | --- | --- |
|  |  |  |  |  |  | 95% Confidence Interval | | |  |  |  |  |  |  |
|  |  | N |  | Mean |  | Lower |  | Upper |  | Median |  | SD |  | IQR |
| age |  | 32 |  | 45.25 |  | 41.96 |  | 48.5 |  | 46.5 |  | 9.14 |  | 12 |
| AUDIT |  | 32 |  | 30.19 |  | 27.98 |  | 32.4 |  | 32 |  | 6.14 |  | 8.25 |
| poms_TMD | | 32 |  | 7.66 |  | 2.82 |  | 12.5 |  | 3.5 |  | 13.41 |  | 12.25 |
| VAS_craving_mm | | 32 |  | 14.19 |  | 8.67 |  | 19.7 |  | 11 |  | 15.29 |  | 19.25 |
| ARRS_Total | | 32 |  | 43.78 |  | 41.14 |  | 46.4 |  | 42 |  | 7.33 |  | 9.75 |
| AB_Responce_time | | 32 |  | 794.43 |  | 744.87 |  | 844 |  | 828.95 |  | 137.48 |  | 203.77 |
| Note. The CI of the mean assumes sample means follow a t-distribution with N - 1 degrees of freedom | | | | | | | | | | |  |  |  |  |
